# The proteasome activator PA200 regulates expression of genes involved in cell survival upon selective mitochondrial inhibition in neuroblastoma cells

**DOI:** 10.1111/jcmm.15323

**Published:** 2020-05-05

**Authors:** Abdennour Douida, Frank Batista, Agnieszka Robaszkiewicz, Pal Boto, Azzam Aladdin, Mónika Szenykiv, Rita Czinege, László Virág, Krisztina Tar

**Affiliations:** ^1^ Department of Medical Chemistry Faculty of Medicine University of Debrecen Debrecen Hungary; ^2^ University of Debrecen Doctoral School of Molecular Medicine Debrecen Hungary; ^3^ Department of Biochemistry and Molecular Biology Faculty of Medicine University of Debrecen Debrecen Hungary; ^4^ Department of General Biophysics Faculty of Biology and Environmental Protection University of Lodz Lodz Poland; ^5^ Stem Cell Differentiation Laboratory Department of Biochemistry and Molecular Biology University of Debrecen Debrecen Hungary

**Keywords:** cell death, cell viability, mitochondrial inhibitors, neuroblastoma, promoter binding, proteasome activator PA200

## Abstract

The conserved Blm10/PA200 activators bind to the proteasome core and facilitate peptide and protein turnover*.* Blm10/PA200 proteins enhance proteasome peptidase activity and accelerate the degradation of unstructured proteasome substrates. Our knowledge about the exact role of PA200 in diseased cells, however, is still limited. Here, we show that stable knockdown of PA200 leads to a significantly elevated number of cells in S phase after treatment with the ATP synthase inhibitor, oligomycin. However, following exposure to the complex I inhibitor rotenone, more PA200‐depleted cells were in sub‐G1 and G2/M phases indicative of apoptosis. Chromatin immunoprecipitation (ChIP) and ChIP‐seq data analysis of collected reads indicate PA200‐enriched regions in the genome of SH‐SY5Y. We found that PA200 protein peaks were in the vicinity of transcription start sites. Gene ontology annotation revealed that genes whose promoters were enriched upon anti‐PA200 ChIP contribute to the regulation of crucial intracellular processes, including proliferation, protein modifications and metabolism. Selective mitochondrial inhibitors induced PA200 redistribution in the genome, leading to protein withdrawal from some gene promoters and binding to others. Collectively, the results support a model in which PA200 potentially regulates cellular homeostasis at the transcriptional level, in addition to its described role as an alternative activator of the proteasome.

## 1 INTRODUCTION

Eukaryotic cells utilize the proteasome, one of the major proteolytic machineries, to degrade proteins. The proteasome is localized in the nuclei and cytoplasm of eukaryotic cells.^1^ The proteasome maintains protein homeostasis by eliminating damaged, misfolded or unneeded proteins. Many cellular events are regulated by specific and timely substrate degradation; therefore, proteasome activity is indispensable for the maintenance of cellular homeostasis.^1^

The activity of the proteasome is tightly regulated. The proteasome is composed of the core particle (CP or 20 S proteasome) and the proteasomal regulatory particles/activators. Currently, three types of proteasome activators have been described: the conserved regulatory particle (RP/19S/PA700),^2^, ^3^ which requires ATP and ubiquitin for substrate processing and degradation; members of the PA28 activator family, present in higher eukaryotes^4^; and the conserved PA200 activator (orthologue of the yeast protein Blm10).^5^, ^6^ PA28 activators and PA200/Blm10 do not require ubiquitin and energy to stimulate proteasomal activity.

PA200 is an evolutionarily conserved protein mainly localized in the nucleus. PA200 is a monomeric protein and contains several HEAT repeats. The Blm10/PA200 activators form hybrid complexes with the proteasome core, where one cap is represented by the 19S and the other end of the core is capped by Blm10/PA200.^6^, ^7^ Blm10/PA200 proteins enhance the peptidase activity of the proteasome^5^, ^6^, ^8^, ^9^ and expedite the degradation of unstructured proteasome substrates, including tau, Dnm1 and acetylated histones.^9^, ^10^, ^11^ Proteasome inhibition is clinically effective in treating certain cancers.^12^, ^13^ A new generation of proteasome inhibitors, including ixazomib, is effective antitumour drugs for the treatment of neuroblastomas in combination with or without doxorubicin.^14^

Previously, miR‐29b (a microRNA that regulates gene expression) replacement by synthetically engineered miRs was shown to reduce the peptidase activity of the proteasome in myeloma cells. The *PSME4* gene, which encodes for PA200, is targeted by miR‐29b, resulting in enhancement of the antimyeloma activities of bortezomib.^15^ Lovastatin, a drug used to treat hypercholesterolemia, increases miR‐29b, resulting in a reduction in PA200.^16^ Furthermore, PA200 is involved in DNA repair and maintenance of genomic stability through enhanced post‐glutamyl cleavage by proteasomes.^5^, ^7^ PA200, together with the core proteasome, accumulates on chromatin following exposure of cells to radiation, independent of the stage of cell cycle arrest.^17^ Additional studies suggest that Blm10/PA200 specifically targets core histones to promote acetylation‐dependent histone degradation by the proteasome, thereby regulating DNA repair mechanisms.^11^, ^18^

Previously, we demonstrated that the proteasome activator, Blm10, is crucial for regulating the proteasomal degradation of the mitochondrial fission protein, Dnm1, in yeast, especially when cells are exposed to oxidative stress.^10^ In addition, many studies report that mitochondrial dysfunction induced by mitochondrial toxins, such as rotenone and oligomycin, can reduce ATP production in neuroblastoma cells and enhance cell migration and invasion in lung cancer cells.^19^, ^20^ Moreover, rotenone induces pathological features, similar to neurodegenerative Parkinson's disease (PD), in neuroblastoma cells.^21^, ^22^ The link between proteasome activity and mitochondrial dysfunction in neurodegenerative diseases is discussed in many studies.^23^, ^24^, ^25^ However, the roles of the proteasome activator PA200 in cell function and diseases have not been elucidated. A study recently demonstrated that PA200 is a negative regulator of human myofibroblast differentiation, partially independent of TGF‐β1 signalling. It was shown that PA200 is up‐regulated in myofibroblasts of fibrotic lungs revealing its role in disease for the first time.^26^ The objective of the present study was to investigate the role of PA200 in the maintenance of neuroblastoma cellular homeostasis, especially when cells are challenged by mitochondrial toxins including rotenone, the agent that reproduces PD.

Our findings demonstrate that PA200 prevents sub‐G1 and G2/M accumulation after complex I inhibition by rotenone. Interestingly, PA200 decreases S phase accumulation after ATP synthase inhibition by oligomycin. Using ChIP‐seq analysis, we show that PA200 is a chromatin component and mitochondrial status defines PA200 association and distribution in the genome of SH‐SY5Y neuroblastoma cells. Finally, we report that PA200 regulates the expression of genes and proteins involved in cell proliferation, cell cycle and cell death in response to mitochondrial toxins. These PA200‐mediated changes in gene and protein expression are dependent on the selective mitochondrial inhibitor.

## 2 MATERIALS AND METHODS

All materials were purchased from Sigma‐Aldrich unless specified otherwise.

### 2.1 Cell culture

Human SH‐SY5Y (European Tissue Culture) cells were maintained in DMEM with high glucose, supplemented with 10% foetal bovine serum (FBS), 2 mmol/L L‐glutamine and 1× (vol/vol) antibiotic‐antimycotic (Gibco, Thermo Fisher Sci, Waltham, MA, USA), at 37°C in a 5% CO_2_ incubator. After generating the stable *PMSE4*/PA200‐depleted human SH‐SY5Y cells using lentiviral technology, the cells were maintained under 1.25 µg/mL puromycin selection. HEK293T cells were cultured in DMEM supplemented with 10% foetal bovine serum (FBS), 2 mmol/L L‐glutamine and 1× (vol/vol) antibiotic‐antimycotic (Gibco) at 37°C in a 5% CO_2_ incubator.

### 2.2 RNA extraction and cDNA reverse transcription

Total RNA was extracted using Extrazol (BioConnect) following the manufacturer's protocol. Samples were treated with DNase I for 15 minutes at room temperature in DNA digestion buffer before the reverse transcription (Zymo Research, Irvine, CA, USA). To perform cDNA synthesis, a High‐Capacity cDNA Reverse Transcription Kit (Applied Biosystems, Foster City, CA, USA) was used to reverse transcribe 1 µg total RNA with random primers. The quality of cDNA was checked by loading 1 µL sample on a 1% agarose gel.

### 2.3 Quantitative real‐time PCR

Real‐time PCR was performed with a LightCycler 480 Thermocycler (Roche) using SYBR *Premix Ex Taq* II from Takara (Clontech) according to the manufacturer's protocol. Cycling conditions are as follows: Stage 1: initial denaturation 95°C for 30 seconds, 1 cycle; Stage 2: PCR 95°C for 5 seconds and 60°C for 30 seconds, 40 cycles; and Stage 3: melt curve analysis 95°C for 0 seconds, 65°C for 15 seconds and 95°C for 0 seconds, cooling 50°C for 30 seconds, 1 cycle. Threshold values (*C*_t_ values) for all replicates were normalized to GAPDH and/or actin. Each of the biological replicates contained three technical replicates for each gene in each sample. To compare the effect of PA200 depletion and the effect of various treatments, 2^−ΔΔCt^ values were calculated to obtain fold expression levels.^27^ The primer list is provided in the Table S2.

### 2.4 Sulphorhodamine B assay

The sulphorhodamine B (SRB) assay, to determine cell viability upon drug treatment, is based on the measurement of cellular protein content. The SRB assay was performed as described by Vichai and Kirtikara.^28^ Cell viability was calculated as follows: % cell viability = Absorbance sample/Absorbance negative control or untreated × 100.

### 2.5 LDH assay

To assess necrosis, a CytoScan LDH Cytotoxicity Assay Kit (Cat.# 786‐210, G‐Biosciences) was used according to the manufacturer's instructions. Briefly, we quantitatively measured the released LDH with a coupled enzyme reaction that converts tetrazolium salt into formazan. The resulting formazan absorbs maximally at 492 nmol/L.

### 2.6 Cell cycle assay

Cell cycle analysis was performed by flow cytometry according to the manufacturer's (Abcam, Cambridge, UK) protocol with a slight modification; 10 µg/mL propidium iodide (PI) was used instead of 50 µg/mL (Sigma, St. Louis, MO, USA). Data acquisition was performed with a NovoCyte 3000 flow cytometer (ACEA Bioscience, Inc). Data were analysed with FlowJo software. After doublet discrimination based on SSC‐W and SSC‐H signals, 25 000 events/sample were analysed, and then, cell cycle analysis was performed on the PI‐A parameter.

### 2.7 Chromatin immunoprecipitation

For each ChIP experiment, chromatin was prepared from SH‐SY5Y cells grown as a monolayer on a plate in the following treatment conditions: DMSO control, 10 µmol/L rotenone, 3 µmol/L oligomycin or 100 nM antimycin A for 3 hour. Cells were cross‐linked using formaldehyde at a final concentration of 1%. After incubation for 10 minutes at room temperature, glycine was added at a final concentration of 0.125 mol/L to quench the cross‐linking. The sample was incubated for 10 minutes at room temperature, pelleted by centrifugation at 400 × *g* for 2 minutes at 4°C, washed two times in cold PBS and resuspended in lysis buffer (1% Triton, 0.1% SDS, 150 mmol/L NaCl, 2 mmol/L EDTA, 1 mmol/L EGTA, 20 mmol/L Tris [pH 8.0]) (200 μL/10 mg chromatin). The sample was then sonicated according to the manufacturer's protocol using the Diagenode Bioruptor Twin (twice for 5 cycles, 30‐s on/off and maximum level). The samples were centrifuged for 2 minutes at 10,000 × *g*, and the supernatant was used for subsequent chromatin IP using antibodies against the proteasome activator 200 kDa protein (PSME4 antibody, PA1‐1961, Thermo Fisher).

For each IP, 200 µL of chromatin was diluted in immunoprecipitation (IP) wash buffer 1 (1% Triton, 0.1% SDS, 150 mmol/L NaCl, 2 mmol/L EDTA, 1 mmol/L EGTA, 20 mmol/L Tris [pH 8.0], 2 μg/μL bovine serum albumin [BSA] and complete protease inhibitor) and incubated with the antibody overnight while rotating at 4°C. Protein A beads were added, and samples were incubated for 6h at 4°C while rotating. The beads were washed twice at 4°C with IP wash buffer 1, once with IP wash buffer 2 (1% Triton, 0.1% SDS, 500 mmol/L NaCl, 2 mmol/L EDTA, 1 mmol/L EGTA, 20 mmol/L Tris [pH 8.0]), once with IP wash buffer 3 (0.25 M LiCl, 1% NP‐40, 1% deoxycholic acid, 1 mmol/L EDTA, 0.5 mmol/L EGTA, 10 mmol/L Tris [pH 8.0]) and twice with Tris‐EDTA buffer, all at 4°C. DNA‐protein complexes were eluted with 400 μL of elution buffer (1% SDS and 0.1 mol/L NaHCO_3_) and decross‐linked by adding NaCl to a final concentration of 0.2 mol/L, followed by shaking overnight at 65°C. DNA was purified using a PCR Clean‐Up Kit from Qiagen and analysed by Chip‐Seq.

### 2.8 ChIP‐seq analysis

ChIP‐seq analysis was performed in Galaxy version 18.01.rc1^29^ using data released by NextSeq 500 System from Illumina (Center for Clinical Genomics and Personalized Medicine, Core Facility, University of Debrecen) for anti‐PA200 pull‐downs and publicly available data from the GEO database (SRR1631233, BioProject PRJNA264969) for anti‐H2K27ac pull‐downs in control (DMSO‐treated) SH‐SY5Y. FASTQ format files were unified to Sanger FASTQ encoding with FASTQ Groomer.^30^ Reads were aligned to the Human Genome (v 19) using Map with Bowtie for Illumina.^31^ ChIP‐seq peaks were called in MACS^32^ with p value cut‐off for peak detection set at 10^‐3^.

PA200 distribution around the transcription start site (TSS) was visualized using compute Matrix/plot Profile (deepTools^33^) with score file: PA200 reads in bigWig format and regions to plot: UCSC Main on Human: gtexGene in BED format. Gene promoters enriched in PA200 and H3K27ac were identified by returning intersects of PA200/H3K27ac peaks (in bed) and genomic regions ±2000 bp centred on TSSs (overlapping intervals of both data sets for at least 1 bp). To compare PA200 occurrence in the genome between control and mitochondrial inhibitor‐treated cells, read depth (bedtools MultiCovBed, mapped reads as BAM to count) was calculated for a set of genomic regions (merged peak intervals as bed for all considered samples). Venn diagrams were created in InteractiVenn (http://www.interactivenn.net/) from generated gene lists.^6^ Enriched gene ontology terms (GO) were derived using PANTHER Classification System (http://pantherdb.org/tools/compareToRefList.jsp; test type—Fisher's exact; reference list—Homo sapiens (all genes in the database) with no correction).

### 2.9 Human phospho‐kinase array

Control cells and shPA200 neuroblastoma cells were treated with vehicle and rotenone and then lysed. Phospho‐kinases were detected according to the manufacturer's protocol (Cat. #ARY003B, R&D Systems). The human phospho‐kinase array simultaneously detects the relative site‐specific phosphorylation of 43 kinases and 2 related total proteins. The levels of phosphorylated protein are assessed using phospho‐specific antibodies and chemiluminescent detection. Images were taken by ChemiDoc Imager, and signal intensity was analysed using Image Lab software.

### 2.10 SDS‐PAGE and Western blot

The cells were rinsed with 1x PBS and lysed in RIPA buffer (50 mmol/L Tris‐HCl, 150 mmol/L NaCl, 0.5% Na‐deoxycholate, 2 mmol/L EDTA, 1% NP‐40, 50 mmol/L NaF) supplemented with a protease inhibitor cocktail (1 mmol/L PMSF, 1 mmol/L benzamidine and 1× EDTA‐free protease inhibitor cocktail) (cOmplete tablets Mini‐EDTA‐free, Roche, Germany). Bradford assay was used to determine protein concentration (Quick Start^TM^ Bradford; # 500‐0205, Bio‐Rad laboratory, USA) according to the manufacturer's instructions. Proteins (30 µg/well) from total cell lysates were separated by SDS‐PAGE. Proteins were transferred to nitrocellulose membranes (0.45 µm NCAmersham, Germany) and were subjected to immunodetection. The Western blotting luminol reagent (#sc‐2048, Santa Cruz Biotechnology, Dallas, TX, USA) was used to visualize the membrane‐bound peroxidase. The images were taken using a ChemiDoc Imager, and signal intensity was analysed using Image Lab software. The antibody list is provided in the Supplementary Material SM1.

### 2.11 Statistical analysis

Data from each experiment were summarized with the mean and standard deviation (SD) of n ≥ 3 experiments. Statistical analyses were performed with one‐way or two‐way ANOVA tests followed by Tukey's multiple comparison test when applicable using GraphPad Prism v8.2.1. Statistical significance was determined as *P < 0.05, **P < 0.01, ***P < 0.001 and ****P < 0.0001.

## 3 RESULTS

### 3.1 Loss of PA200 sensitizes cells to rotenone, but not oligomycin‐induced cell death

**FIGURE 1:**
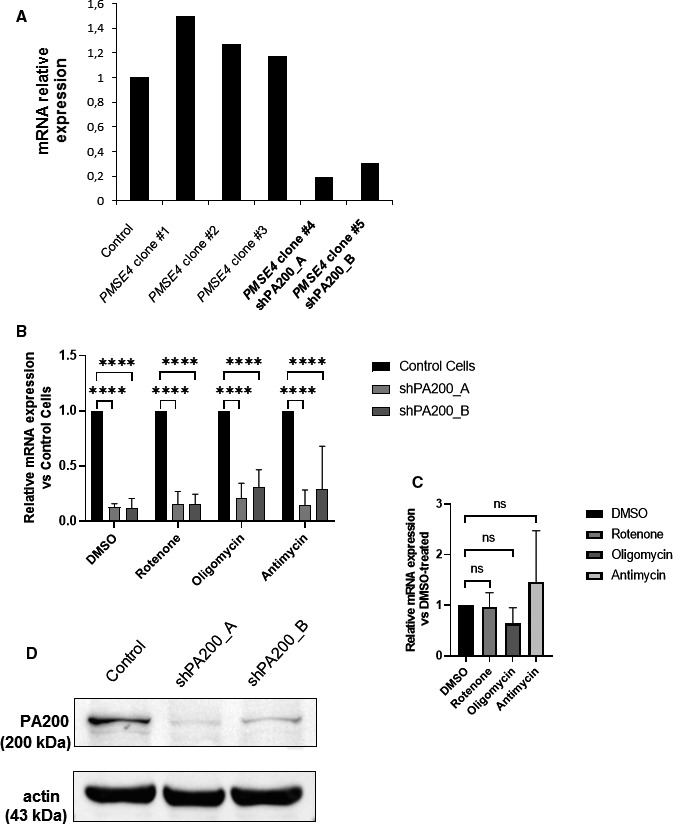
Generation of a stable PA200‐depleted neuroblastoma cell line using lentiviral technology. Down‐regulation of the expression of PSME4/PA200 in SH‐SY5Y human neuroblastoma cell line was achieved using lentiviral technology. To produce the virus, HEK293T cells were transfected with a mixture of packaging‐enveloping vectors and the plasmids pGIPZ‐GFP containing the shRNA target sequences (PMSE4 clones #1, #2, #3, #4 and #5). Cells stably expressing empty pGIPZ‐GFP were used as a control. Transduction of SH‐SY5Y neuroblastoma cells was conducted using 8 µg/mL polybrene, and cells were maintained in 1.25 µg/mL puromycin for selection. Puromycin was removed 24 h before performing every experiment. (A)‐(B) PA200‐depleted cells and corresponding control cells were treated with vehicle (DMSO) or mitochondrial inhibitors (10 µmol/L rotenone, 3 µmol/L oligomycin, 100 nmol/L antimycin A) for 24 h. The qPCR analysis showed extensive silencing of PA200 in PMSE4 clone #4 and PMSE4 clone #5 later referred to as shPA200_A and shPA200_B, respectively. Results are presented as the mean ± SD of n = 4 independent experiments. Statistical analysis was performed by ANOVA using GraphPad Prism v8.2.1. (*P < 0.05, **P < 0.01, ***P < 0.001, and ****P < 0.0001). (C) The control cell line was treated with vehicle (DMSO) or mitochondrial inhibitors (10 µM rotenone, 3 µmol/L oligomycin, 100 nmol/L antimycin A) for 24 h. A qPCR analysis was performed to check the relative mRNA expression of *PMSE4*. Results are presented as the mean ± SD of n = 4 independent experiments. Statistical analysis was performed by ANOVA using GraphPad Prism v8.2.1. (D) Total cell lysates from shPA200 cells (shPA200_A, shPA200_B) and control cells were prepared, and equal protein amounts of each sample were separated by SDS‐PAGE to analyse the protein level of PA200. Actin was used as the loading control

To validate the potential role of PA200 (gene name: *PMSE4*) in cellular homeostasis, shRNAs were utilized to create stable gene knockdowns in SH‐SY5Y neuroblastoma cells (See supporting information and methods SM2 and SM3 for detailed description and Table S1 for the shRNA clone list). The knockdown efficiency was analysed by qRT‐PCR and Western blotting. Successful knockdown was achieved in two clones, *PMSE4* clone 4 (referred to as shPA200_A, fold change (absolute value) FC (abs) = 6, 289; FC = −6, 289) and clone 5 (shPA200_B, FC (abs) = 4, 087, FC = −4, 807)) (Figure 1A,B). As a control, we used SH‐SY5Y cells stably expressing the pGIPZ‐GFP (referred to as control). In addition, in every qPCR experiment, we also assessed the level of PA200 transcript in the stably depleted cells to confirm the stable down‐regulation. To perform our experiments, we chose clone 4 shPA200‐depleted cell line, because PA200 protein expression was almost completely abolished by the gene depletion (Figure 1D) and the gene depletion was not affected by treatment with mitochondrial inhibitors (Figure 1B). We also evaluated the effect of mitochondrial inhibitors on the mRNA expression of *PMSE4* in control cells, and the mitochondrial toxins do not significantly alter the expression of *PMSE4* (Figure 1C).

We and other investigators have previously shown that the loss of *BLM10* (the yeast orthologue of *PA200)* results in a yeast phenotype with dysfunctional mitochondria, especially when cells are exposed to stress.^10^, ^34^, ^35^ Therefore, to investigate whether PA200 knockdown enhances the susceptibility to mitochondrial agents, we treated cells for 24 hour with three mitochondrial inhibitors. The mitochondrial inhibitors affected different elements of the electron transport chain: (a) Rotenone selectively inhibited complex I of the mitochondrial respiratory chain, (b) oligomycin blocked ATP synthase, and (c) antimycin A inhibited the complex III.

**FIGURE 2:**
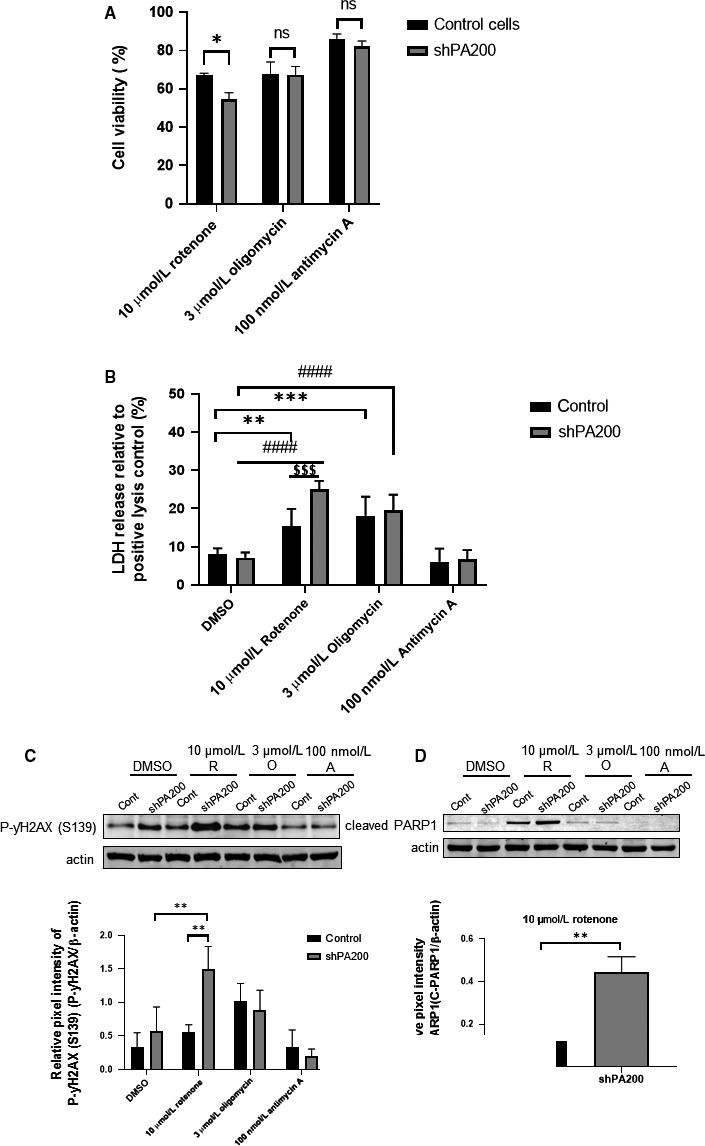
PA200 affects cell viability after treatment with mitochondrial inhibitors. PA200‐depleted cells and corresponding control cells were treated with vehicle (DMSO) or mitochondrial inhibitors for 24 h. (A) Cell cytotoxicity was assessed using a sulphorhodamine B assay. Before the assay, 5000 cells were plated in each well of a 96‐well plate. On the following day, cells were treated for 24 h with either DMSO or mitochondrial inhibitors at the indicated concentration in DMEM complete media without puromycin. Results are presented as the mean ± SD of six independent experiments. Statistical analysis was performed by ANOVA using GraphPad Prism v8.2.1. (*P < 0.05, **P < 0.01, ***P < 0.001, and ****P < 0.0001). (B) Assessment of necrosis using the LDH assay. Cells were treated with DMSO, 10 µmol/L rotenone, 3 µmol/L oligomycin or 100 nmol/L antimycin A. Results are presented as the mean ± SD of three independent experiments. Puromycin was removed 24 h before performing every experiment. Statistical analysis was performed by ANOVA using GraphPad Prism v8.2.1. (*P < 0.05, **P < 0.01, ***P < 0.001, and ****P < 0.0001). (C)‐(D) Apoptotic markers were verified by SDS‐PAGE and Western blot in control and shPA200‐depleted cells. Control and shPA200 neuroblastoma cell lines were incubated with vehicle (DMSO), 10 µmol/L rotenone (R), 3 µmol/L oligomycin (O) or 100 nmol/L antimycin A (A) for 24 h. Total cell lysates were separated by SDS‐PAGE, transferred to nitrocellulose membranes and subjected to Western blot analysis to check the level of phosphorylated γH2AX (C) and cleaved PARP1 (D). Actin was used as an internal loading control. Densitometry was performed on three independent biological replicates (C‐D lower panels) with Image Lab software. Data are presented as mean values ± SD and represent 3 independent experiments. Statistical analysis was performed by ANOVA using GraphPad Prism v8.2.1. (*P < 0.05, **P < 0.01, ***P < 0.001, and ****P < 0.0001)

We determined the effects of PA200 silencing on cellular viability using a SRB assay in the presence and absence of mitochondrial inhibitors (Figure 2A). No significant changes in cell viability were observed in vehicle‐treated cells depleted of PA200 compared with control vehicle‐treated cells (data not shown). However, after 10 µmol/L rotenone treatment, shPA200 cells showed a significant decrease in viability compared with the rotenone‐treated control (Figure 2A). Treatment with 3 μmol/L oligomycin did not cause a significant difference in viability when cells were depleted of PA200 compared with the oligomycin‐treated control cell line (Figure 2A). We also measured the cytotoxic effect of 100 nmol/L antimycin A, but did not detect any significant changes compared with antimycin A‐treated control cells (Figure 2A).

Necrosis was determined 24 hour after treatment with mitochondrial inhibitors by measuring the cellular release of lactate dehydrogenase (LDH). The PA200‐depleted cells showed similar LDH release as the control cell line after treatment with oligomycin or antimycin A (Figure 2B). In contrast, treatment with 10 µmol/L rotenone resulted in a significant increase in LDH release in PA200‐depleted cells compared with rotenone‐treated control cells (Figure 2B). To summarize, cells depleted of PA200 responded to mitochondrial stress with either increased necrosis or an unaltered rate of cell survival in an inhibitor‐dependent manner compared with their respective control. We also checked apoptosis in both cell lines upon selective mitochondrial inhibition. We measured protein levels of known apoptotic markers by Western blot analysis. γH2AX is a histone that is rapidly phosphorylated at Ser139 following DNA damage in response to apoptotic signals.^36^ Cleaved PARP1 is considered to be a hallmark of apoptotic cell death.^37^ As Figure 2C,D shows, we detected a significantly higher level of phosphorylated γH2AX and cleaved PARP1 in shPA200‐depleted cells following rotenone treatment compared with rotenone‐treated control cells. The phosphorylated γH2AX level was increased, but comparable in both cell lines after oligomycin exposure. However, 3 μmol/L oligomycin treatment did not affect PARP1 cleavage. Taken together, these data suggest that silencing of PA200 sensitizes cells to rotenone‐induced cellular death.

### 3.2 Cell cycle shift in PA200‐depleted cells in a mitochondrial inhibitor‐dependent manner

**FIGURE 3:**
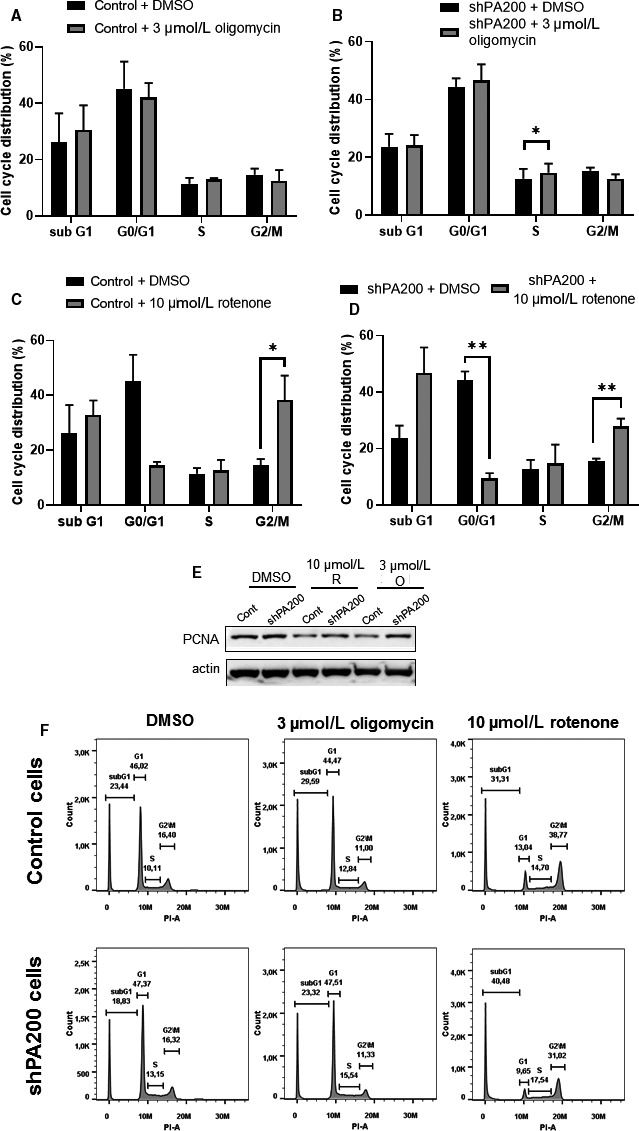
Cell cycle change in PA200 knockdown cell line after selective exposure to mitochondrial inhibitors. Cell cycle distributions were measured by flow cytometry after 24‐h treatment with DMSO (vehicle), 3 µmol/L oligomycin (A)‐(B) or 10 µmol/L rotenone (C)‐(D), respectively. Results are displayed as mean ± SD of 3 independent experiments, and statistically significant differences were calculated using RM ANOVA test (*P < 0.05, **P < 0.01). (E) The effects of PA200 on cell proliferation upon selective mitochondrial inhibition were analysed using SDS‐PAGE and Western blot to detect the proliferative cell nuclear antigen protein (PCNA). Control and shPA200 neuroblastoma cell lines were incubated with vehicle (DMSO), 10 µmol/L rotenone (R) or 3 µmol/L oligomycin (O) for 24 h. Total cell lysates were separated by SDS‐PAGE, transferred to nitrocellulose membranes and subjected to Western blot analysis. Actin was used as an internal loading control. (F) Representative image of cell distribution of cell cycle by flow cytometry analysis

Follow‐up experiments were performed after rotenone and oligomycin exposure only, because antimycin A did not show any significant effects in shPA200 cells at the concentrations used. (We checked 100, 150 and 200 nmol/L antimycin A on cell viability as shown in Figure 2 and Figure S3A,B, respectively.) The distribution of cell cycle phases in control and PA200‐depleted cells was analysed by flow cytometry. Quantitative analysis revealed that a significantly elevated number of PA200‐depleted cells were in the S phase after oligomycin treatment compared with DMSO‐treated PA200‐depleted cells indicative of maintained, however possibly delayed DNA replication (Figure 3A,B,F). To assess overall cell proliferation changes, we checked the level of proliferating cell nuclear antigen (PCNA). The PCNA protein plays an essential role in DNA replication as an axillary protein for Polδ, the enzyme responsible for the replication of chromosomal DNA.^38^ As Figure 3E demonstrates, the level of PCNA in mitochondrial toxin‐treated PA200‐depleted cells was higher, compared with mitochondrial toxin‐treated control cells, suggesting maintained proliferation.

Rotenone treatment resulted in a reduced G0/G1 population with a concomitant increase in cells in the G2/M phase in both cell lines (Figure 3C,D). These results indicate that rotenone has an anti‐proliferative effect, which was associated with an arrest in the G2/M phase of the cell cycle in both cell lines, in addition to an increase in cells in the sub‐G1 phase after depletion of PA200. These data suggest that the stable down‐regulation of PA200 might alter cell cycle progression upon selective mitochondrial inhibition.

### 3.3 PA200 is a chromatin component in SH‐SY5Y neuroblastoma cells

A previous study revealed that the deletion of *BLM10*, the yeast orthologue of PA200, resulted in the down‐regulation of genes encoding for proteins required for proper chromosome organization, assembly, function, repair and progression throughout the cell cycle.^39^ Therefore, we speculated that the responses to mitochondrial inhibitors in the PA200‐depleted cells might be associated with overall transcriptional changes upon loss of PA200 in mammalian cells as well. We have been suggested that PA200 might be involved in the regulation of cellular stability through its direct or indirect (as a co‐factor) binding to the promoter regions of certain relevant genes. Recently, it was shown that PA200 participates in the degradation of acetylated core histones during DNA damage response, for example after UV radiation.^11^, ^18^ Based on a crystal structure study, Blm10, the yeast orthologue of PA200, contains a bromodomain BRD‐like (BRDL) region that recognizes and binds acetylated regions of proteins.^35^ PA200 is predicted to have a similar region at aa1650‐1738 with Phe^1676^/ Asn^1716^ Phe^1717^. However, the BRDL regions of Blm10/PA200 do not share any sequence homology similarities with known BRDs. Nevertheless, it was demonstrated that in vitro the BRD‐like regions of Blm10 and PA200 could bind acetyl‐histones. However, post‐translational modifications of histones might be required to help the binding.^11^ A recent characterization of a fully recombinant 20S‐PA200 structure also revealed that the residues are indeed neighbours, but the side chain of Phe1676 is buried in the protein structure and oriented away from those of Asn1716 and Phe1717. The authors did not identify bromodomains in the PA200 structure. They speculate based on their cryo‐EM map of non‐protein densities in their 20S‐PA200 that inositol phosphates binding to PA200 might be correlated with PA200’s role in the degradation of acetylated histones.^40^

**FIGURE 4:**
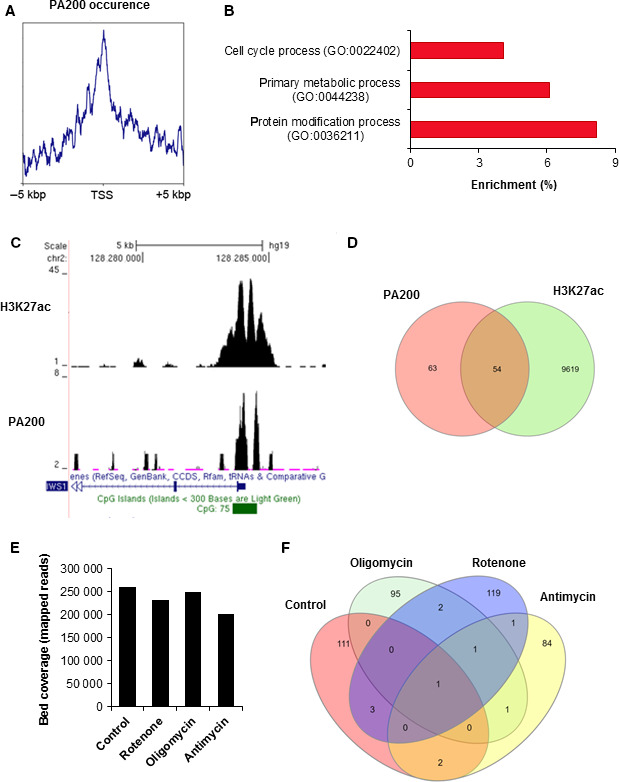
PA200 occurrence in the genome of SH‐SY5Y responds to cell treatment with mitochondria‐impairing agents. (A) PA200 distribution around gene transcription start sites (TSS) was monitored by analysing mapped ChIP‐seq reads with deepTools. (B) and (C) Peak calling in MACS revealed PA200 presence at gene promoters and their biological function, analysed with Amigo2 (B), disclosed crucial cellular processes. (C) Some PA200‐positive gene promoters were simultaneously characterized by histone acetylation (H3K27ac) that marks actively transcribed genes. (D) Venn diagrams display PA200‐enriched active (+H3K27ac) and inactive (‐H3K27ac) gene promoters. (E) PA200 association with chromatin after cell treatment with 10 μmol/L rotenone, 3 μmol/L oligomycin and 100 nmol/L antimycin A was monitored and quantified by bedCoverage. (F) Alterations in the occupancy of gene promoters by PA200 in untreated cells and cells challenged with mitochondria‐impairing agents were compared by Venn diagrams

To verify our hypothesis that PA200 might bind to promoters, we conducted PA200 chromatin immunoprecipitation followed by next‐generation sequencing (ChIP‐seq). Bioinformatic analysis of collected reads clearly indicated PA200‐enriched regions in the genome of SH‐SY5Y (Table S4). To determine the possible functionality of PA200 on chromatin, we monitored the distribution of chromatin‐bound PA200 and found that protein peaks were located in the vicinity of transcription start sites (TSSs) (Figure 4A). These data suggest that PA200 may act as a transcriptional regulator. However, further studies are needed to confirm such an idea and disclose the mechanism underlying the molecular role of PA200 in transcriptional regulation. Gene ontology annotation revealed that genes that were significantly enriched in PA200 contribute to the regulation of crucial intracellular processes, including proliferation, protein modifications and metabolism (Figure 4B; Table S5). Thus, we speculate that PA200 might be a chromatin‐associated regulator of cellular physiology.

To test whether PA200 is bound to promoters of actively transcribed or repressed/silent genes, PA200 peaks were collated with H3K27ac peaks in the Genome Browser (example in Figure 4C). As PA200 was found in both H3K27ac‐positive and H3K27ac‐negative promoters, the quantification of PA200 occurrence vs acetylation status was displayed in a Venn diagram (Figure 4D). Such an approach disclosed the lack of mutual interdependence between PA200 association with gene promoters and gene transcription status (protein peaks were comparably distributed on acetylated and deacetylated promoters).

### 3.4 Mitochondrial status defines PA200 association and distribution in the genome

Bearing in mind that the PA200 yeast orthologue Blm10 is required for the maintenance of mitochondrial function,^10^, ^35^ we tested whether challenging mitochondria with rotenone, oligomycin or antimycin A affects PA200 association with chromatin and the distribution of PA200 in the genome. Two (rotenone and antimycin A) out of the three tested agents caused slight eviction of PA200 from chromatin (Figure 4E; Table S6), suggesting that mitochondrial status affects PA200 interaction with DNA. Furthermore, all studied compounds induced PA200 redistribution in the genome leading to protein withdrawal from some gene promoters and binding to others (Figure 4F; Tables [Link], [Link], [Link]). Our data suggest that mitochondrial inhibition may alter gene transcription by shifting PA200 on chromatin. However, additional studies are required to verify such a hypothesis.

### 3.5 PA200 regulates transcription of genes involved in cell cycle progression and apoptosis

**FIGURE 5:**
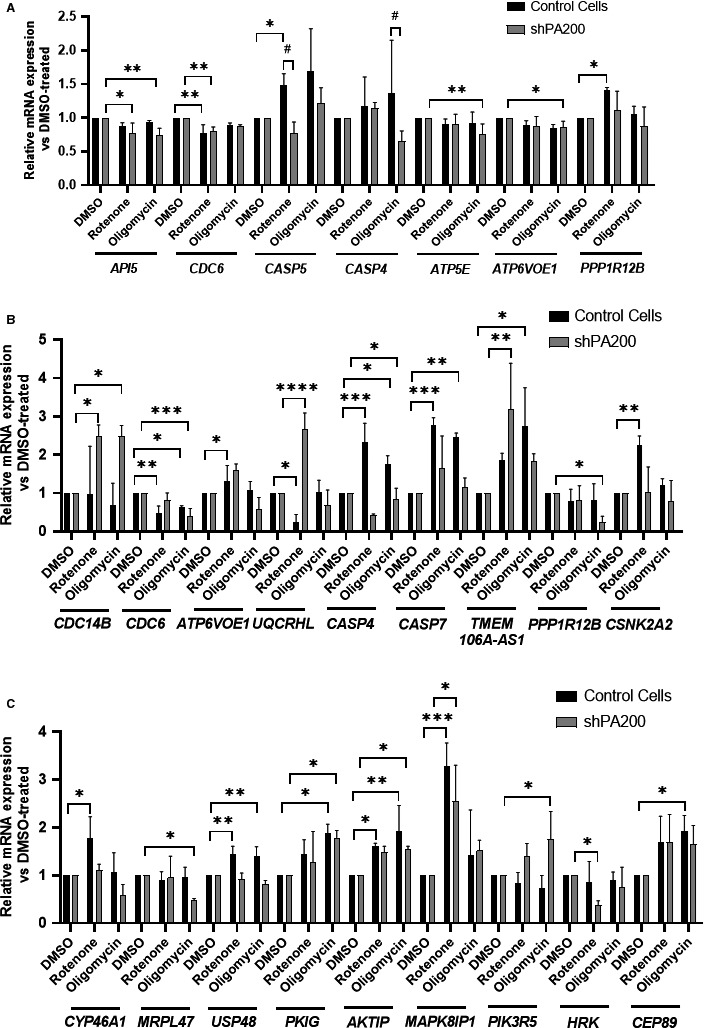
Validating expression of PA200 target genes. (A) Expression of PA200 target genes was validated by quantitative real‐time PCR after 3‐h treatment with selective mitochondrial inhibitors. Before RNA extraction, shPA200 and control cells were incubated with vehicle (DMSO) or the mitochondrial inhibitors, 10 µmol/L rotenone and 3 µmol/L oligomycin, for 3 h. The mRNA expression of selective mitochondrial inhibitor‐treated (10 µmol/L rotenone and 3 µmol/L oligomycin) control cells and shPA200 cells was normalized to DMSO‐treated control and DMSO‐treated shPA200 cells, respectively. Puromycin was removed 24 h before performing every experiment. Data are presented as the mean ± SD of three separate experiments. Statistical analysis was performed by ANOVA using GraphPad Prism v8.2.1. (*P < 0.05, **P < 0.01, ***P < 0.001, and ****P < 0.0001). (B**)‐(**C) Expression of PA200 target genes was validated by quantitative real‐time PCR after 24 h treatment with selective mitochondrial inhibitors. Before RNA extraction, shPA200 and control cells were incubated with vehicle (DMSO) or the mitochondrial inhibitors, 10 µmol/L rotenone and 3 µmol/L oligomycin, for 24 h. The mRNA expression of selective mitochondrial inhibitor‐treated (10 µmol/L rotenone and 3 µmol/L oligomycin) control cells and shPA200 cells was normalized to DMSO‐treated control and DMSO‐treated shPA200 cells, respectively. Puromycin was removed 24 h before performing every experiment. Data are presented as the mean ± SD of three separate experiments. Statistical analysis was performed by ANOVA using GraphPad Prism v8.2.1. (*P < 0.05, **P < 0.01, ***P < 0.001, and ****P < 0.0001)

Next, we analysed the expression patterns of certain genes whose promoters showed enrichment on the anti‐PA200 ChIP with and without selective mitochondrial inhibition. These genes are involved in cell cycle progression, apoptosis and mitochondrial function. We analysed their expression by quantitative real‐time PCR. Figure 5A demonstrates gene expression patterns after 3‐hr treatment with selective mitochondrial inhibitors, whereas Figure 5B,C show significant gene expression changes following 24‐hr exposure, to demonstrate the dynamic changes in gene expression. The fold gene expression change following exposure to mitochondrial toxins was compared with the respective DMSO‐treated cell line. For example, the promoters of *CASP5* and *CASP4* were enriched upon rotenone and oligomycin treatment, respectively (Tables S7 and S8). The qPCR data of the 3‐hour treatment (Figure 5A) already show up‐regulation of both genes after the respective treatment; however, loss of PA200 results in a significant down‐regulation of both genes following the corresponding mitochondrial inhibitor treatment. The 24‐hour treatment demonstrates similar gene expression patterns for certain genes (Figure 5B‐C), including *CASP4, CASP7* (promoter enrichment after rotenone exposure, Table S7), *PPP1R12B* (enriched after oligomycin exposure, Table S8) and *CSNK2A2* (enriched after rotenone treatment, Table S7). The genes were significantly up‐regulated in control cells following treatment; however, the effect was lost upon depletion of PA200. Taken together, the fact that several gene promoters were specifically bound by PA200 (ChIP‐seq data) and this binding was validated by mRNA expression levels of these genes demonstrated that this cohort of validated promoters was bona fide targets of PA200 in human neuroblastoma cells.

### 3.6 PA200 knockdown leads to reduced c‐jun following rotenone administration

**FIGURE 6:**
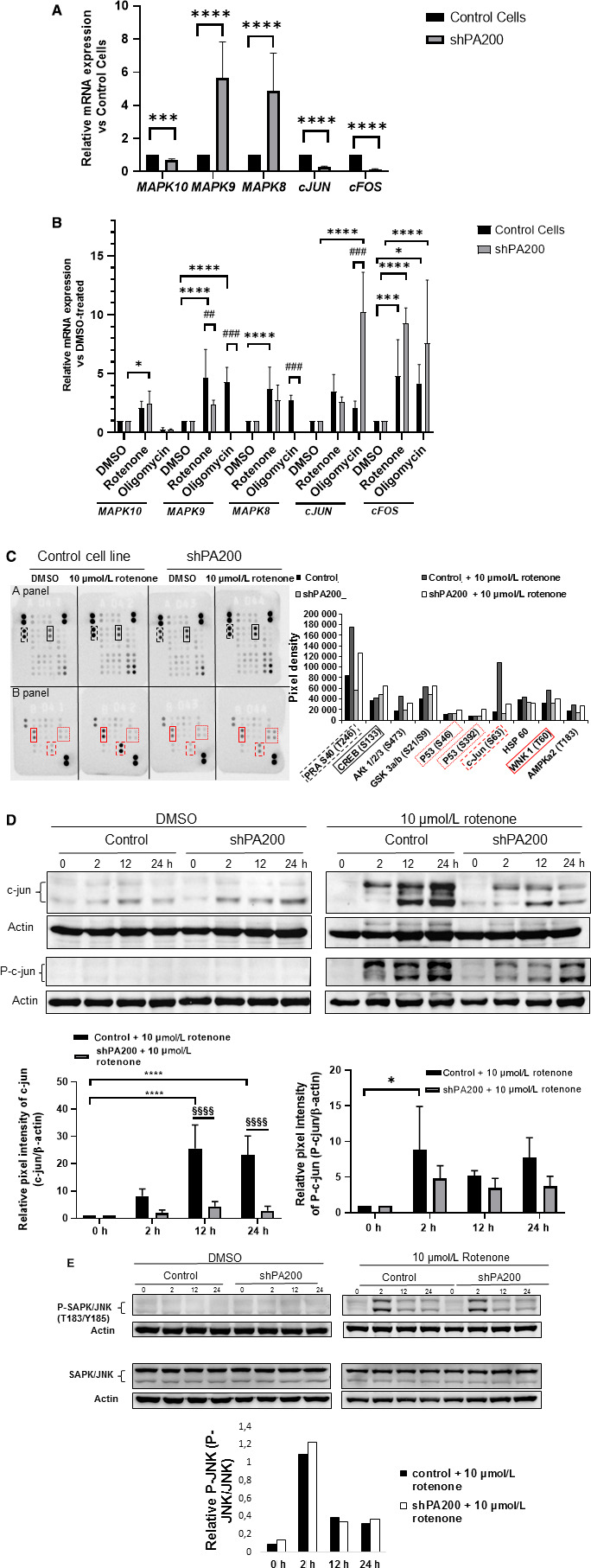
PA200 regulates the expression of genes and proteins implicated in the control of cell proliferation and cell stress. The mRNA level of cellular proliferation and stress genes was analysed using quantitative real‐time PCR analyses. Before RNA extraction, shPA200 and control cells were incubated with vehicle (DMSO) or the mitochondrial inhibitors, 10 µmol/L rotenone and 3 µmol/L oligomycin, for 24 h. (A) The mRNA levels of vehicle‐treated shPA200 were normalized to vehicle‐treated controls. (B) The mRNA expression of selective mitochondrial inhibitor‐treated (10 µmol/L rotenone and 3 µmol/L oligomycin) control cells and shPA200 cells was normalized to DMSO‐treated control and DMSO‐treated shPA200 cells, respectively. Puromycin was removed 24 h before performing every experiment. Data are presented as the mean ± SD of four separate experiments. Statistical analysis was performed by ANOVA using GraphPad Prism v8.2.1. (*P < 0.05, **P < 0.01, ***P < 0.001, and ****P < 0.0001). (C) Control and shPA200 neuroblastoma cell lines were incubated with vehicle (DMSO) or 10 µmol/L rotenone for 24 h. Total cell lysates obtained from control and shPA200 cells after 24‐h treatment were used to analyse phosphorylation profiles of kinases using the human phospho‐kinase array. Labels: kinase array panel A: xxx PRAS S40 and xxx CREB; kinase array panel B: xxx WNK 1, xxx c‐Jun and xxx p53. Representative bars of the pixel intensity of dots in the human phospho‐kinase array. For data analyses, Image Lab 5.2.1 software was used. (D) Confirmation of the effects of PA200 on the c‐Jun‐JNK pathway by SDS‐PAGE and Western blot. Control and shPA200 neuroblastoma cell lines were incubated with vehicle (DMSO) or 10 µmol/L rotenone for the indicated time‐points. Total cell lysates (30 µg protein) obtained from control and shPA200 cells at indicated time‐points were separated by SDS‐PAGE, transferred to nitrocellulose membranes and subjected to Western blot analysis. Actin was used as an internal loading control (right panel). Densitometry for c‐Jun and p‐c‐Jun after rotenone treatment was performed on three independent biological replicates of control and shPA200 cells (left panel). Data are presented as mean values ± SD of 3 independent experiments. Statistical analysis was performed by ANOVA using GraphPad Prism v8.2.1. (*P < 0.05, **P < 0.01, ***P < 0.001, and ****P < 0.0001). (E) Activation of the SAPK/JNK pathway was analysed using SDS‐PAGE and Western blot to check total and phosphorylated SAPK/JNK protein levels. Control and shPA200 neuroblastoma cell lines were incubated with vehicle (DMSO) or 10 µmol/L rotenone for the indicated time‐points. Total cell lysates (30 µg protein) obtained from control and shPA200 cells at indicated time‐points were separated by SDS‐PAGE, transferred to nitrocellulose membranes and subjected to Western blot analysis. Actin was used as an internal loading control. The SAPK/JNK antibody detects endogenous levels of total JNK1, JNK2 or JNK3 protein. Western blot analysis was performed in n = 3 independent experiments

One known pathway for rotenone‐induced cell death is through activation of the JNK pathway and by phosphorylation of c‐Jun in SH‐SY5Y neuroblastoma cells.^41^, ^42^ We saw an increased sub‐G1 cell population in shPA200 cells indicative of apoptosis. Thus, based on our bioinformatic data and the previous literature, we validated the expression of genes whose promoters were found enriched in the anti‐PA200 ChIP, and/or are involved in the JNK pathway, including MAPK10 (enriched in vehicle‐treated cells, Table S4) and MAPK8IP1 (enriched in oligomycin‐treated samples, Table S). We compared mRNA expression in control and PA200‐depleted cells in the presence or absence of selective mitochondrial inhibitors. We found that the mRNA expression of *MAPK8* (MAPK8/JNK1) and *MAPK9* (MAPK9/JNK2) was among those strongly up‐regulated, whereas *JUN* (c‐jun), *FOS* (proto‐oncogene c‐fos) and *MAPK10* (MAPK10/JNK3) were strongly down‐regulated in vehicle‐treated PA200‐depleted cells compared with vehicle‐treated control cells (Figure 6A upper panel).

Rotenone treatment led to significant up‐regulation of most of the examined genes compared with vehicle treatment, however to a different extent in control vs shPA200 cells (Figure 6B). All *MAPK*s were down‐regulated and *JUN* and *FOS* were up‐regulated in shPA200 cells after oligomycin treatment compared with vehicle‐treated shPA200 cells, whereas control cells showed up‐regulation in all measured genes except for *MAPK10* after oligomycin exposure (Figure 6B).

To determine whether phosphorylation of specific kinases and transcription factors, especially c‐Jun, was affected by PA200 depletion, we first performed a human phospho‐kinase array. Most of the kinases showed similar phosphorylation levels in PA200‐silenced cells after rotenone administration compared with rotenone‐treated control cells (Figure 6C, See Table S3 for co‐ordinate references). As expected, we observed elevated c‐Jun phosphorylation in control cells after rotenone treatment (Figure 6C, right panel). Surprisingly, the phosphorylation of c‐Jun at Ser63 was severely reduced in PA200‐depleted cells following rotenone administration (Figure 6C, left panel).

To confirm the diminished phosphorylation of c‐Jun in PA200‐depleted cells upon rotenone treatment, we also performed Western blot analysis of total cell lysates at the indicated time‐points after rotenone administration. Total c‐Jun and phospho‐c‐Jun were barely detectable in both vehicle‐treated cell lines (Figure 6D, right panel). Rotenone (10 µmol/L) resulted in an increase in total c‐Jun at 2 hour in both control and shPA200 cell lines (Figure 6D, left upper panel). However, this increase was significantly less in PA200‐depleted cells (Figure 6D, left upper panel). In rotenone‐treated control cells, phosphorylation of c‐Jun appeared at 2 hour and increased over the 24‐hour treatment (Figure 6D, left lower panel). However, in PA200‐depleted cells, c‐Jun was weakly phosphorylated 2 hour after rotenone treatment, and the weak phosphorylation of c‐Jun was maintained in the later stages of treatment (Figure 6D, left lower panel).

The activity of c‐Jun is also stimulated by JNK phosphorylation in neuroblastoma cells.^43^ Thus, we evaluated the role of JNKs. Cells were treated with vehicle control or 10 µmol/L rotenone for the indicated times. We assayed the activities of JNKs by Western analysis using antibodies that recognize dual‐phosphorylated and activated JNKs and endogenous levels of total JNK1 (also known as MAPK8), JNK2 (MAPK9) and JNK3 (MAPK10). JNK phosphorylation was apparent 2 hour after treatment with rotenone and was maintained at a lower level over time in both control and shPA200 cells, but not in DMSO‐treated cells. (Figure 6E). We concluded that the JNK pathway was comparably activated upon rotenone treatment in both cell lines and the reduced phospho‐c‐Jun following rotenone exposure resulted from a reduced c‐Jun pool in cells depleted of PA200, concomitant with both gene and protein expression data, and not from the inactivation of the JNK pathway.

## 4 DISCUSSION

In this study, we have presented data indicating that PA200 is required for the regulation of neuroblastoma cell survival in response to selective mitochondrial stress. We chose neuroblastoma cells, for several reasons. First, proteasome inhibition by second‐generation proteasome inhibitors sensitizes neuroblastoma cells to apoptosis; therefore, they are potential therapeutic targets for treating neuroblastoma patients.^44^ Second, rotenone treatment of neuroblastoma cells results in similar pathological features to Parkinson's disease and is a good model to study neurodegeneration.^21^, ^22^ Third, the role of the proteasome in many diseases, including neurodegenerative diseases, is a well‐studied field.^23^, ^24^ However, the role of PA200 is unknown in diseases and may represent a good target for new therapeutic approaches. We have shown that the proteasome activator, PA200, is recruited to the chromatin and is associated with promoters of genes involved in the cell cycle, primary metabolism and protein modification processes. We monitored the distribution of chromatin‐bound PA200 and found that protein peaks were centred in the vicinity of transcription start sites (TSSs) and that this recruitment is dependent on the conditions that cause mitochondrial stress.

We evaluated the effect of PA200 depletion on cell death and cell proliferation after exposure to mitochondrial toxins. The mitochondrial inhibitors used in this study are toxins, known to trigger cell death in several cell types, including neuroblastoma cells. Inhibition of ATP synthesis by oligomycin induces apoptotic cell death in pro‐lymphocyte B cells.^45^ In our cell model, death of PA200‐depleted cells was comparable to control cells upon oligomycin exposure. However, cell cycle analysis indicated significantly increased cell population in the S phase, indicative of possible DNA replication delay upon PA200 depletion following oligomycin treatment. In addition, Western blot analysis of PCNA showed an increased level of protein after oligomycin treatment in the PA200‐depleted cell line compared with oligomycin‐treated control cells, indicating that PA200 might contribute to oligomycin‐induced cell death.

Previous literature suggests that PA200 accumulates on chromatin in response to ionizing radiation (IR).^46^ The authors demonstrated that PA200 deficiency might result in increased chromosome aberrations even without exposing HeLa cells to IR. The authors proposed that PA200 is required for correct post‐glutamyl proteasomal activity and the maintenance of genomic stability during the IR‐induced cellular response. Another study revealed that the deletion of *BLM10*, the yeast orthologue of PA200, resulted in the down‐regulation of genes encoding for proteins required for proper chromosome organization, assembly, function, repair and progression throughout the cell cycle.^39^ We speculated that depletion of PA200 might sensitize neuroblastoma cells to selective mitochondrial inhibitor‐induced cell death, and this response might be associated with transcriptional changes.

In our cell model, ChIP and ChIP‐seq analysis indicated PA200‐enriched regions in the genome of SH‐SY5Y. Moreover, the studied mitochondrial inhibitors induced PA200 redistribution in the genome leading to protein withdrawal from some gene promoters and binding to others. For example, ChIP‐seq data revealed that PA200 binds to the promoter region of *ATP5E*, the mitochondrial ATP synthase F1 subunit epsilon. However, PA200 was evicted from the *ATP5E* promoter after oligomycin treatment (Table S4). Havlícková et al^47^ demonstrated that the silencing of the ATP5E subunit in HEK cells resulted in decreased activity and content of the ATP synthase complex. In addition, our gene expression data show significant down‐regulation of *ATP5E* in PA200‐silenced cells following oligomycin treatment (Figure 5A). Thus, it is tempting to speculate that the rate of ATP synthesis might be altered by PA200 and that inhibiting mitochondrial ATP production might result in elevated glycolytic ATP production in PA200‐depleted cells and altered cell metabolism. In addition, after oligomycin exposure, PA200 binds to the promoters of *CASP4* and *MAPK8* *IP1*, whose levels seem to influence the cell survival response (Table S8).^48^ In addition, *CASP4* mRNA expression was significantly down‐regulated in PA200‐silenced cells after oligomycin treatment compared with vehicle‐treated PA200‐depleted cells (Figure 5A,B). Thus, depending on the status of binding or eviction of PA200 to/from specific promoters after oligomycin exposure, cells might go through an adaptive mechanism.

Rotenone, on the other hand, can induce apoptosis through elevated mitochondrial ROS production in HL‐60 cells^49^ or SH‐SY5Y cells by activating the JNK/Jun pathway.^41^, ^42^ Cell cycle analysis after rotenone treatment showed an elevated sub‐G1 population and G2/M cell cycle arrest in shPA200 cells indicative of apoptosis. Furthermore, apoptotic markers were elevated in PA200‐depleted cells compared with their respective controls. Interestingly, cells depleted of PA200 exhibited strong down‐regulation of *JUN,* and significantly reduced c‐jun level and c‐jun phosphorylation after rotenone treatment. Although inactive or reduced levels of c‐jun in shPA200 cells can still support cell proliferation similar to control cells, cells were not protected from rotenone‐induced apoptosis. Similar to a published study.^50^ c‐jun phosphorylation might not be required for proliferation in cells depleted of PA200. However, c‐jun phosphorylation is required for protection against rotenone. Moreover, after rotenone exposure, we found the promoters of *CASP5*, *CASP7* and *CSNK2A2* enriched in the anti‐PA200 ChIP (Table S7). Caspases 5 and 7 are known mediators of apoptosis, whereas casein‐kinase 2 regulates cell cycle progression, apoptosis and several transcription factors including c‐jun. Casein‐kinase 2 up‐regulation correlates with poor cancer prognosis in numerous cancers.^51^ Stable depletion of PA200 without treatment dramatically down‐regulated genes that are critical for maintaining balanced cell proliferation and integrity suggesting a vulnerability of cells lacking PA200 (Figure S1). Furthermore, stable depletion of PA200 selectively sensitized cells to different mitochondrial inhibitors (Figure S2A,B).

Our ChIP‐seq evidence indicates that PA200 binds to promoters in a mitochondrial agent‐dependent manner; thus, PA200 might be a key player in the response to diverse mitochondrial stimuli at the transcriptional level. Based on our data presented here, the mutual interdependence between PA200 and histone acetylation seems to go beyond the simple occupation of transcriptionally active promoters. PA200 was equally found at the acetylated and deacetylated promoters. Furthermore, gene transcription was both up‐ and down‐regulated by PA200. The specificity of how this regulation is achieved and whether PA200 directly binds to DNA or through specific transcription factors requires further investigation. As our data suggest, different stimuli induce altered binding of PA200 to transcriptional targets. These targets await further molecular validation.

## CONFLICT OF INTEREST

The authors have no competing financial or non‐financial interests to declare.

## AUTHORS' CONTRIBUTION

KT and RCz performed lentiviral silencing; FB, KT, AA and AD carried out real‐time qPCR; AD, MSz and RCz performed cellular assays; FB, AR and KT involved in ChIP and ChIP‐seq analysis; PB and AD involved in FACS analysis. LV contributed essential reagents or tools; FB, KT and PB involved in statistics; AR, FB, KT, PB and AD analysed the data; and KT prepared the original draft of the manuscript, KT, FB and AR reviewed and edited the manuscript, KT supervised the study and acquired funding. All authors read and approved the version of the manuscript to be published.

## Supporting information

Fig S1

Fig S2

Fig S3

Table S4

Table S5

Table S6

Table S7

Table S8

Table S9

Supplementary Material

Supplementary Material

## Data Availability

The data that support the findings of this study are available from the corresponding author upon reasonable request.
