# Supplementary figures and images for: The proteasome activator PA200 regulates expression of genes involved in cell survival upon selective mitochondrial inhibition in neuroblastoma cells
Source: J Cell Mol Med. 2020 May 5;24(12):6716–30. doi: 10.1111/jcmm.15323 (PMC7299700; doi:10.1111/jcmm.15323)

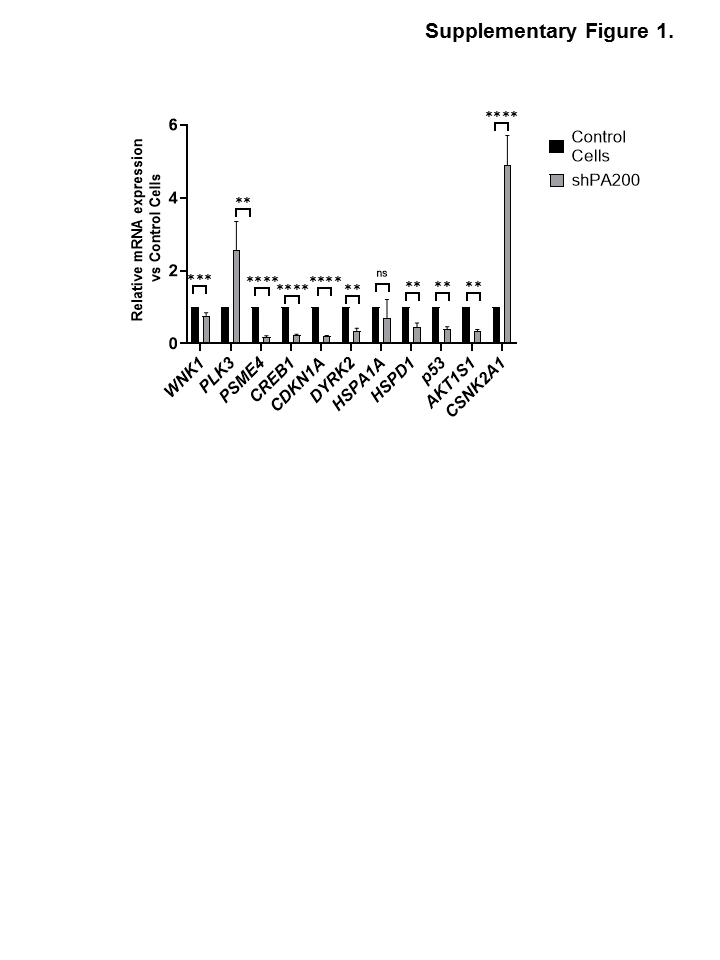

Supplement: Supplementary file 1 — Fig S1 [file JCMM-24-6716-s001.TIF]

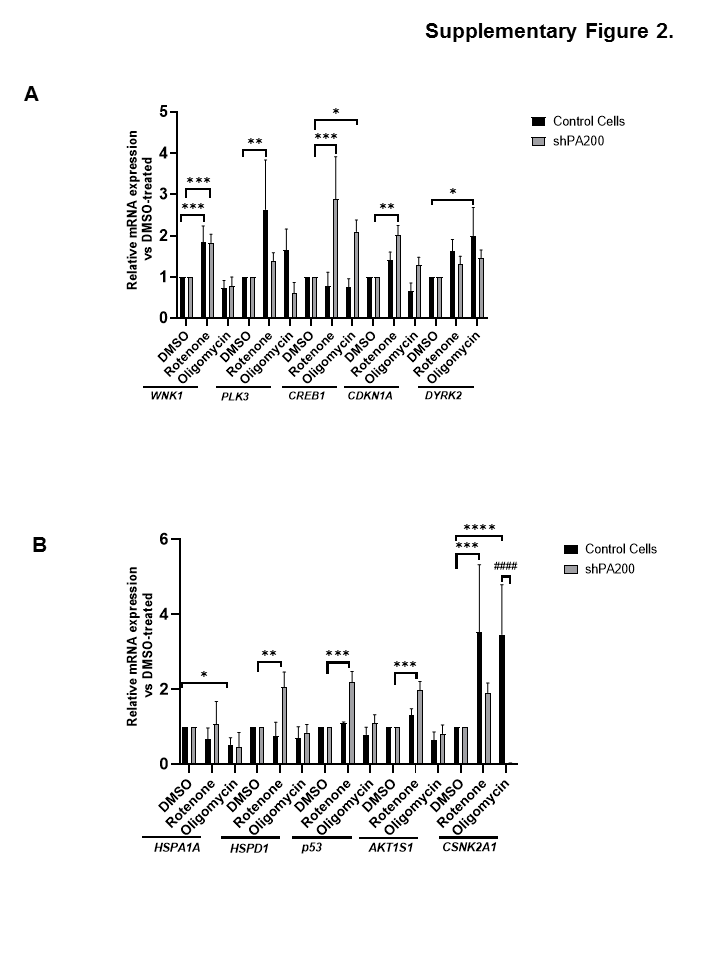

Supplement: Supplementary file 2 — Fig S2 [file JCMM-24-6716-s002.TIF]

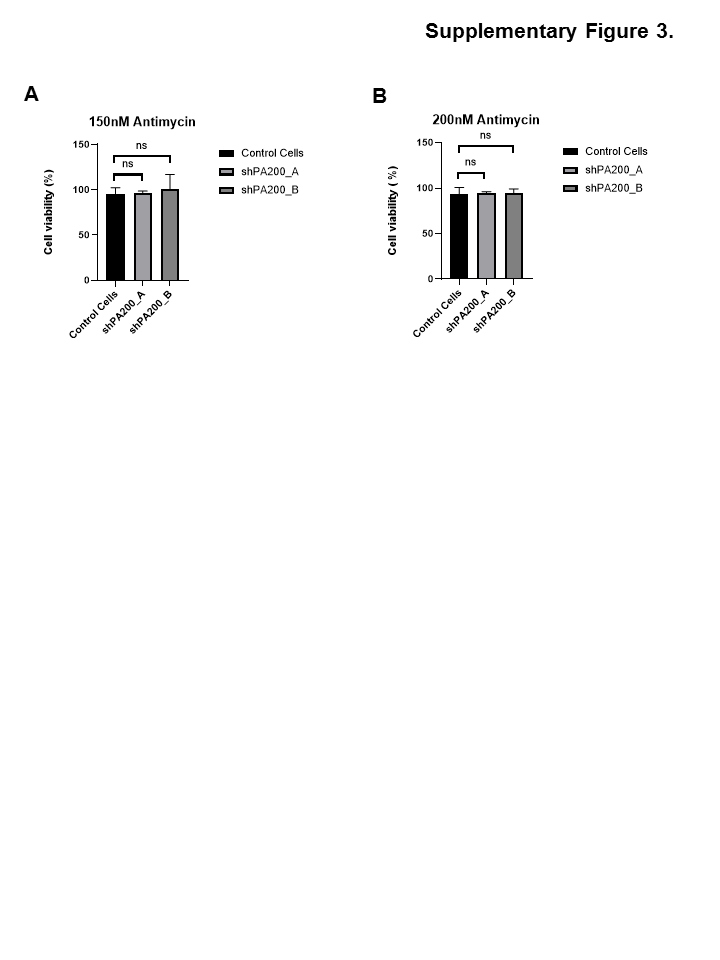

Supplement: Supplementary file 3 — Fig S3 [file JCMM-24-6716-s003.TIF]
