# Supplementary material for: The proteasome activator PA200 regulates expression of genes involved in cell survival upon selective mitochondrial inhibition in neuroblastoma cells
Source: J Cell Mol Med. 2020 May 5;24(12):6716–30. doi: 10.1111/jcmm.15323 (PMC7299700; doi:10.1111/jcmm.15323)
Supplement: Supplementary file 10 — Supplementary Material [file JCMM-24-6716-s010.docx]

**Supporting Information**

**Supplementary Table 1. shRNA clone list used in this study**

| **Gene ID** | **Gene symbol** | **Species** | **Vector** | **Marker** | **Sense strand sequence** | **Start** | **End** | **Target** |
| --- | --- | --- | --- | --- | --- | --- | --- | --- |
| 23198 | PSME4 | Human | pGIPZ | GFP | CCCAGCGATGTGTTGCAGA | 4180 | 4198 | CDS |
| 23198 | PSME4 | Human | pGIPZ | GFP | AGGTCTGTCTTCTACGTTT | 1808 | 1826 | CDS |
| none | GIPZ empty vector |  | pGIPZ | GFP | Empty pGIPZ vector  (no shRNA) |  |  |  |
| none | GIPZ non-targeting |  | pGIPZ | GFP | Non-silencing shRNA sequence CTCGCTTGGGCGAGAGTAA |  |  |  |

**Supplementary Table 2. Primers list**

| **Gene symbol** | **Species** | **Forward sequence** | **Reverse sequence** |
| --- | --- | --- | --- |
| PSME4 | human | ATGGAGAGTGCCTGAACTATTG | GTAGGTCAGCACACTTCCTATTC |
| PSME4a | human | GACCCTGGTTCTGTAGGAGATA | GGGAACATCGTAAGGACTAGAAAG |
| PSME4b | human | CCCTGGAAAAGCCATCAATA | TCCTGTTGGCGTTTAATTCC |
| PSME4isoform1 | human | GCACATTGGAGCAAACAAGA | ACCATGCGACCTGCTACTCT |
|  | | | |
| JUN | human | CCCCAAGATCCTGAAACAGA | CCGTTGCTGGACTGGATTAT |
| FOS | human | CCGGGGATAGCCTCTCTTAC | GTGGGAATGAAGTTGGCACT |
| TP53 | human | CCTCCCATGTGCTCAAGACT | GCTCGACGCTAGGATCTGAC |
| MAPK8 | human | TGTGGAATCAAGCACCTTCA | GTTTTCCTTGTAGCCCATGC |
| MAPK9 | human | GGGAACACCATCAGCAGAGT | GTATGGGTGACGCAGAGCTT |
| MAPK10 | human | TGGAAGTGGGAGACTCAACC | TGGTTCTGAAAGGGTCTGCT |
| PLK3 | human | GACCTGAGCTGGAGATGCTG | GCTGCCAGTCTCTGTGTCAG |
| CREB1 | human | GTGTTACGTGGGGGAGAGAA | GGGCTAATGTGGCAATCTGT |
| WNK1 | human | ATCGAAGAGCTGGAGACCAA | TGGGATGCTGAAGACCTTTT |
| DYRK2 | human | GCCAGAAGTAGCAGCAGGAC | GAAGCCTGAAGCTGACGAAC |
| CSNK2A1 | human | TGCCAGATCTCCAAACATCA | TTACCTCGGCCTAATTTTCG |
| CSNK2A2 | human | GTCTACGCCGAGGTGAACAG | TGGTGATATTAATGGCCTCAAA |
| CDKN1A | human | GGAAGACCATGTGGACCTGT | GGATTAGGGCTTCCTCTTGG |
| HSPA1A | human | CGACCTGAACAAGAGCATCA | AAGATCTGCGTCTGCTTGGT |
| HSPD1 | human | CACAGTCTTTCGCCAGATGA | ACTTCCCCAACTCTGCTCAA |
| AKT1S1 | human | GAGGATGAGCCCACAGAGAC | GGGGTCTGACTCACAGAAGG |
| API5 | human | CTCACCATGCCGACAGTAGA | CAGCCAATTCTGGAAAATGC |
| CDC6 | human | AGTTTGTTCAGGGGCTTGTG | CGAGACAGCTTCCTTTTTGG |
| CDC14B | human | CCTCCATGAAGCGGAAAAG | TGATGCACTCTTTGGTCTGC |
| CASP5 | human | ACGGATCAAAAGTCGACCAG | GATCAGGGCCTTGTCTTCAA |
| CASP4 | human | TCTCACCTGTTGGAATGCAC | TGACCCGAACTTTGTCTTCA |
| CASP7 | human | GAGCGACGGAGAGAGACTGT | GAAGCACAAATCCCACTGCT |
| ATP5E | human | CCTACTGGAGACAGGCTGGA | TTTTACGTTGCTGCCAGAAG |
| ATP6VOE1 | human | TTGTGATGAGCGTGTTCTGG | CTTCTTCCTCAAGGCCAATG |
| PPP1R12B | human | ACCAGTTCCCACCTGCTATG | ATTGATGCCTGTGCCTCTTC |
| UQCRHL | human | ATGCGAGCAGTTGGAGAAAT | TCTGATGCCCAGATGATGAA |
| TMEM106A-AS1 | human | TCAATCCTGTCCTCCAAACC | ATGAGAGCCACCAACTGCTT |
| CYP46A1 | human | GGCGCTGACAGCTGAGTC | AGGGGAGGTGTCCTAGAAGG |
| MRPL47 | human | TGGCCCTTCTTTGTAGGAGA | ACTTTTTCTTGCCCCCAGTT |
| USP48 | human | AGCACATCGAGACCGCTTAC | TCTCACAGTTGGGATCATCG |
| PKIG | human | CATCCAGGGAGACTCAGAGG | GCCTTCTTGGACAAGGTCAG |
| AKTIP | human | GCCGAAGACAGAAGATTTGG | GAAGGCAGCTGTTTCTTTGG |
| MAPK8IP1 | human | AATGGCGGAGCGAGAAAG | GGACAGGGTGTCTTTGCACT |
| PIK3R5 | human | ACCCGAATCGCAGGGAGT | GGCTGAGGCTGAGTCCAT |
| HRK | human | CAGGCGGAACTTGTAGGAAC | CCCAGTCCCATTCTGTGTTT |
| CEP89 | human | GTCCCTGGCTGTGGATAGAT | TCTCTCTGGAGATGGGTTGG |
| GAPDH | human | GAGTCAACGGATTTGGTCGT | GATCTCGCTCCTGGAAGATG |
| beta- actin | human | GACCCAGATCATGTTTGAGACC | CATCACGATGCCAGTGGTAC |

**
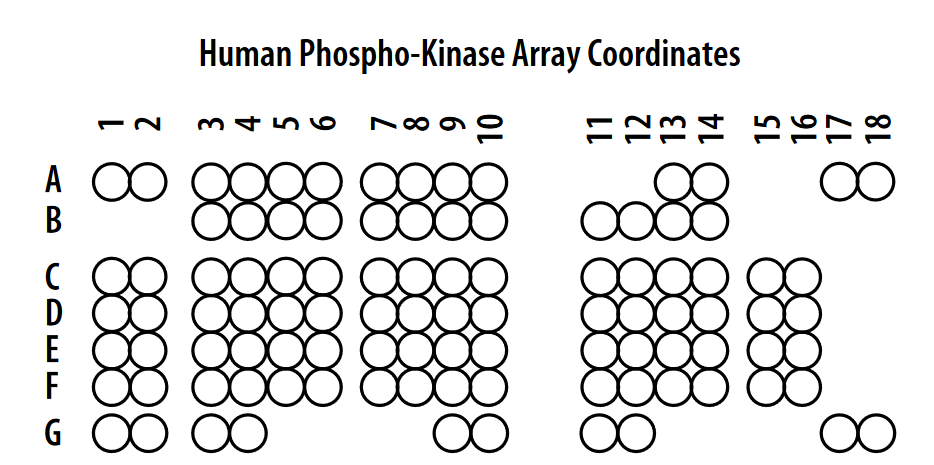
Supplementary Table 3. Human phospho-kinase array coordinates reference**

| **Membrane/Coordinate** | **Target/Control** | **Phosphorylation site** |
| --- | --- | --- |
| A-A1, A2 | Reference Spot | - |
| A-A3, A4 | p38α | T180/Y182 |
| A-A5, A6 | ERK1/2 | T202/Y204, T185/ Y187 |
| A-A7, A8 | JNK 1/2/3 | T183/Y185, T221/ Y223 |
| A-A9, A10 | GSK-3α/β | S21/S9 |
| B-A13, A14 | p53 | S392 |
| B-A17, A18 | Reference Spot | - |
| A-B3, B4 | EGF R | Y1086 |
| A-B5, B6 | MSK1/2 | S376/S360 |
| A-B7, B8 | AMPKα1 | T183 |
| A-B9, B10 | Akt 1/2/3 | S473 |
| B-B11, B12 | Akt 1/2/3 | T308 |
| B-B13, B14 | p53 | S46 |
| A-C1, C2 | TOR | S2448 |
| A-C3, C4 | CREB | S133 |
| A-C5, C6 | HSP27 | S78/S82 |
| A-C7, C8 | AMPKα2 | T172 |
| A-C9, C10 | β-Catenin | -__ |
| B-C11, C12 | p70 S6 Kinase | T389 |
| B-C13, C14 | p53 | S15 |
| B-C15, C16 | c-Jun | S63 |
| A-D1, D2 | Src | Y419 |
| A-D3, D4 | Lyn | Y397 |
| A-D5, D6 | Lck | Y394 |
| A-D7, D8 | STAT2 | Y689 |
| A-D9, D10 | STAT5a | Y694 |
| B-D11, D12 | p70 S6 Kinase | T421/S424 |
| B-D13, D14 | RSK1/2/3 | S380/S386/S377 |
| B-D15, D16 | eNOS | S1177 |
| A-E1, E2 | Fyn | Y420 |
| A-E3, E4 | Yes | Y426 |
| A-E5, E6 | Fgr | Y412 |
| A-E7, E8 | STAT6 | Y641 |
| A-E9, E10 | STAT5b | Y699 |
| B-E11, E12 | STAT3 | Y705 |
| B-E13, E14 | p27 | T198 |
| B-E15, E16 | PLC-γ1 | Y783 |
| A-F1, F2 | Hck | Y411 |
| A-F3, F4 | Chk-2 | T68 |
| A-F5, F6 | FAK | Y397 |
| A-F7, F8 | PDGF Rβ | Y751 |
| A-F9, F10 | STAT5a/b | Y694/Y699 |
| B-F11, F12 | STAT3 | S727 |
| B-F13, F14 | WNK1 | T60 |
| B-F15, F16 | PYK2 | Y402 |
| A-G1, G2 | Reference Spot | -___ |
| A-G3, G4 | PRAS40 | T246 |
| A-G9, G10 | PBS (Negative Control) | -___ |
| B-G11, G12 | HSP60 | - |
| B-G17, G18 | PBS (Negative Control) | -__ |

**Supplementary materials and methods**

**SM1. List of antibodies used in this study for western blot**

rabbit anti-PA200 (1:1000, #NBP2-22236, NovusBio.)

rabbit anti-SAPK/JNK (1:1000, #9252s, Cell Signaling Technology)

rabbit anti-phospho-SAPK/JNK (T183/Y185, 1:1000, #9251s, Cell Signaling Technology)

mouse anti-c-Jun (1:250, #sc-74543, Santa Cruz Biotechnology)

mouse anti-phospho-c-Jun (S63) (1:250, #sc822, Santa Cruz Biotechnology)

goat anti-actin (1:25000, #sc1616, Santa Cruz Biotechnology)

rabbit- anti PCNA (1:1000, # 13110, Cell Signaling Technology)

phosphorylated histone H2AX (4418-APC-100) (TREVIGEN)

rabbit- anti Cleaved PARP (Asp214) (D64E10) mAb #5625 Cell Signaling Technology)-

**SM2. Downregulation of *PSME4*/PA200**

We used lentiviral technology to downregulate the expression of *PSME4*/PA200. Briefly, one day prior to transfection confluent HEK293T cells were seeded to reach 80% confluency the next day in a 24-well plate. The expression plasmids pGIPZ-GFP containing the target sequences and the control plasmids were obtained from the shRNA Facility of the Albert Einstein College of Medicine, Bronx, NY, USA and were provided by Dr. Marion Schmidt. All information on the plasmids is listed in the supplementary material. The packaging and enveloping vectors (HDM-Hgpm2, RC-CMV/Rev, HDM-tat1b, and HDM-VSV-G) were courtesy of Dr. Orsi Giricz (Albert Einstein College of Medicine, Bronx, NY, USA). Transfection was performed using Lipofectamine 3000 (ThermoFisher) according to the manufacturer’s protocol. Four wells were used for each construct. To prepare the HDM-MIX, HDM-Hgpm2, RC-CMV/Rev, HDM-tat1b (25 ng/µl each), and 50 ng/µl of HDM-VSV-G were used. The expression plasmids were used at 100 ng/µl. The HDM-MIX and the expression plasmids were co-transfected at a 1:1 ratio. After 24 and 48 hr, the transfected cells were visualized by fluorescent microscopy to confirm the expression of GFP. Culture media was collected 48 and 96 hr after transfection. Virus-containing media was filtered through 0.45-µm pore filters and immediately used for transduction.

Transduction of human SH-SY5Y neuroblastoma cells was conducted using the following protocol. Cells were incubated with the virus containing antibiotic-free media supplemented with 8 µg/ml polybrene. Cells were monitored every day under a fluorescent microscope to check GFP expression. Selection with 1.25 µg/ml puromycin was started 72 hrs after viral transduction. The puromycin selected and amplified cells were further analyzed by real-time PCR and Western blot to verify the depletion of *PSME4*/PA200.

**SM3. Titration of antibiotic selection**

To generate stable cell lines of transduced target cells, the selection antibiotic was titrated to determine the optimal concentration used for the selection of transduced target cells. SH SY5Y cells were plated to reach 90 % confluency in a 6-well plate. The appropriate complete medium was added containing 0, 0.5, 1, 1.25, 2.5, or 5 µg/ml puromycin. Cells were incubated for 10 days, replacing the selection media every two days. Cells were monitored using a light microscope every day. The lowest puromycin concentration (1.25 µg/ml) that begins to give massive cell death in 7 days and kills all cells within 10 days was used for further selection and to maintain the stable cell line under antibiotic selection pressure.
