# Supplementary material for: The proteasome activator PA200 regulates expression of genes involved in cell survival upon selective mitochondrial inhibition in neuroblastoma cells
Source: J Cell Mol Med. 2020 May 5;24(12):6716–30. doi: 10.1111/jcmm.15323 (PMC7299700; doi:10.1111/jcmm.15323)
Supplement: Supplementary file 11 — Supplementary Material [file JCMM-24-6716-s011.docx]

**Supplementary Figure 1**

The mRNA level of cellular proliferation, apoptotic and stress genes was analysed using quantitative real-time PCR analyses. Prior to RNA extraction, shPA200 and control cells were incubated with vehicle (DMSO) or the mitochondrial inhibitor, 10 µM rotenone and 3 µM oligomycin, for 24 hr. The mRNA levels of vehicle-treated shPA200 were normalized to vehicle-treated controls. Puromycin was removed 24 hr prior to performing every experiment. Data are presented as the mean ± SD of four separate experiments. Statistical analysis was performed by Anova test using GraphPad Prism v8.2.1. (* indicates p < 0.05, ** indicates p < 0.01, *** indicates p < 0.001, **** indicates p < 0.0001).

**Supplementary Figure 2**

(**A and B**) The mRNA expression of selective mitochondrial inhibitor-treated (10 µM rotenone and 3 µM oligomycin) control cells and shPA200 cells were normalized to DMSO treated control and DMSO treated shPA200 cells, respectively. Puromycin was removed 24 hr prior to performing every experiment. Data are presented as the mean ± SD of four separate experiments. Statistical analysis was performed by Anova test using GraphPad Prism v8.2.1. (* indicates p < 0.05, ** indicates p < 0.01, *** indicates p < 0.001, **** indicates p < 0.0001).

**Supplementary Figure 3.**

(**A and B**) PA200-depleted cells and corresponding control cells were treated with vehicle (DMSO) or Antimycin A for 24h. Cell viability was assessed using a sulforhodamine B assay. Prior to the assay, 5, 000 cells were plated in each well of a 96-well plate. On the following day, cells were treated for 24 hr with either DMSO or Antimycin A at the indicated concentration in DMEM complete media without puromycin. Results are presented as the mean ± SD of three independent experiments normalized to vehicle-treated control. Puromycin was removed 24 hr prior to performing every experiment. Statistical analysis was performed by Anova using GraphPad Prism v8.2.1.
